# A Dual-Intein Autoprocessing Domain that Directs Synchronized Protein Co-Expression in Both Prokaryotes and Eukaryotes

**DOI:** 10.1038/srep08541

**Published:** 2015-02-25

**Authors:** Bei Zhang, Madhusudhan Rapolu, Zhibin Liang, Zhenlin Han, Philip G. Williams, Wei Wen Su

**Affiliations:** 1 Department of Molecular Biosciences and Bioengineering, University of Hawaii at Manoa, Honolulu, Hawaii 96822, USA; 2 Department of Chemistry, University of Hawaii at Manoa, Honolulu, Hawaii 96822, USA

## Abstract

Being able to coordinate co-expression of multiple proteins is necessary for a variety of important applications such as assembly of protein complexes, trait stacking, and metabolic engineering. Currently only few options are available for multiple recombinant protein co-expression, and most of them are not applicable to both prokaryotic and eukaryotic hosts. Here, we report a new polyprotein vector system that is based on a pair of self-excising mini-inteins fused in tandem, termed the dual-intein (DI) domain, to achieve synchronized co-expression of multiple proteins. The DI domain comprises an *Ssp* DnaE mini-intein N159A mutant and an *Ssp* DnaB mini-intein C1A mutant connected in tandem by a peptide linker to mediate efficient release of the flanking proteins *via* autocatalytic cleavage. Essentially complete release of constituent proteins, GFP and RFP (mCherry), from a polyprotein precursor, in bacterial, mammalian, and plant hosts was demonstrated. In addition, successful co-expression of GFP with chloramphenicol acetyltransferase, and thioredoxin with RFP, respectively, further substantiates the general applicability of the DI polyprotein system. Collectively, our results demonstrate the DI-based polyprotein technology as a highly valuable addition to the molecular toolbox for multi-protein co-expression which finds vast applications in biotechnology, biosciences, and biomedicine.

Intein is a naturally found protein splicing element that undergoes self-excision from a protein precursor with concomitant joining (splicing) of the flanking protein sequences called exteins. This protein splicing process is autocatalytic and does not require the presence of any exogenous host-specific proteases or co-factors. The discovery of intein has enabled a wide variety of innovative applications including enzyme activation, protein purification, expressed protein ligation, and production of cyclic proteins^1^,^2^,^3^,^4^. In addition to being an effective splicing element, inteins can be engineered to block the splicing activity and promote auto-excision at their N- or C- terminal, by mutating the crucial terminal residues, as well as the extein residues immediately flanking the intein sequence. Here we exploit the hyperactive intein-mediated self cleavage to develop a system for coordinating co-expression of multiple proteins from a single open reading frame (ORF).

Simultaneous and balanced co-expression of multiple proteins enables many important applications in biochemistry, life sciences, and biotechnology. Some notable examples are: (*i*) production of multimeric pharmaceutical proteins such as antibodies and multi-subunit vaccines for disease diagnosis and treatment^5^,^6^,^7^ (*ii*) synthesis of protein complexes for biochemical, biophysical, and structural studies^8^; (*iii*) modification of metabolic pathways^9^; (*iv*) elucidation of how complex metabolic networks are organized and regulated^10^; as well as (*v*) engineering of crops with desirable agronomic traits^11^.

Currently three strategies are most commonly practiced for co-expression of multiple proteins: (*i*) multiple monocistronic expression cassettes on the same or separate vectors; (*ii*) polycistronic vectors based on ribosome binding site (RBS) or internal ribosomal entry site (IRES) for prokaryotic and eukaryotic hosts, respectively; and (*iii*) polyprotein vectors based on foot-and-mouth-disease-virus (FMDV) 2A-like peptide or protease substrate sequence for eukaryotic hosts^12^,^13^. With respect to the use of multiple monocistronic cassettes for co-expression, control over stoichiometric expression of the multiple transgenes cannot be readily achieved even after extensive promoter tuning. Also, driving the expression of each transgene cassette using the same type of promoter does not necessarily result in the same level of expression for each transgene^14^. When linked transgene expression cassettes are used on a single vector, because each gene cassette harbors its own set of regulatory elements, the size of the vector could become quite large especially if the transgene number increases. Such large vector size could substantially attenuate the transformation/transfection efficiency. As for the IRES-mediated translational initiation, it is known to be far less efficient compared with that of the 5′-cap mediated initiation. This leads to highly uneven co-expression, and the efficiency is shown to be cell-type specific^14^. Regarding the FMDV-2A or 2A-like viral peptide sequences, efficacy of protein co-expression is affected by the peptide sequence or protein structure immediately upstream of the 2A motif in a manner that is poorly understood^15^,^16^,^17^. While many examples of successful 2A-mediated co-expression have been reported, there are also cases where a considerable portion of the polyprotein fails to separate into its constituent proteins^15^. Upon co-translational release, the 2A sequence remained on the carboxyl terminus of its upstream protein could cause erroneous subcellular targeting, or may interfere with the folding and function of certain target proteins^12^,^18^. Also notably, 2A- and IRES- based systems only work in eukaryotes.

Owing to the ease of genetic manipulation and ability to produce a large amount of recombinant proteins in a short period of time, *Escherichia coli* is commonly used as a test bed for functional studies of novel engineered proteins or protein complexes. Ultimate validation of the biological function of the protein of interest in a different host such as plant or mammalian cells would be necessary when the primary goal is to use or test the function of the protein in these hosts. It is therefore necessary to establish an efficient multi-protein co-expression platform that can be readily adopted in different hosts.

Here, we report a new polyprotein vector system that is based on a pair of self-excising mini-inteins fused in tandem, termed the dual-intein (DI) domain, to achieve coordinated co-expression of multiple proteins. The DI fusion domain comprises an *Ssp* DnaE mini-intein N159A mutant and an *Ssp* DnaB mini-intein C1A mutant connected in tandem by a peptide linker. This unique fusion domain harnesses the synergy between the N- and C- terminal auto-cleaving activity of DnaE (N159A) and DnaB (C1A) mini-intein variant, respectively, to mediate autocatalytic release of the flanking proteins *in vivo* (Fig. 1a). As intein autoprocessing can proceed efficiently in both prokaryotes and eukaryotes, we envisioned that the DI based polyprotein system can be applicable to a variety of expression hosts. In this study, we investigated the DI system in bacterial, mammalian, and plant hosts, and confirmed its usefulness in all host systems tested.

## Results and discussion

### Designing the dual-intein based polyprotein system for efficient autoprocessing

Efficient autoprocessing (cleaving) of a polyprotein precursor *in vivo* into physically separated constituent proteins is essential in developing a polyprotein-based vector system for coordinating co-expression of multiple proteins from a single ORF. This is achieved here by incorporating engineered intein elements. Generally, intein splicing begins with the N-S/O acyl shift reaction at the amide bond preceding the N-terminal junction with the side chain of the first intein residue (position 1, typically Cys or Ser) (Supplementary Fig. S1). Thereafter, the resulting linear (thio)ester intermediate undergoes trans(thio)esterification by nucleophilic attack from the sulfhydryl or hydroxyl groups on the side chain of the first C-extein residue (position +1, typically Cys, Ser, or Thr) and subsequently forms a branched intermediate. Finally, the branched intermediate is resolved by Asn cyclization of the intein C-terminal to liberate the intein fragment and ligate N- and C-exteins via a (thio)ester bond, which can be spontaneously rearranged into a more stable amide bond^19^.

**Figure 1:**
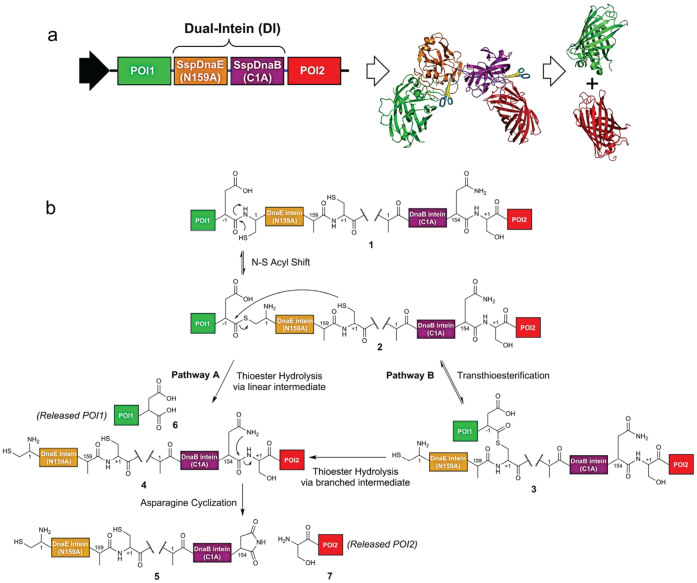
Synchronized co-expression of multiple proteins using the DI-based polyprotein vector system. (a) Organization of the DI-based polyprotein ORF, and autoprocessing of the polyprotein precursor into two separate proteins of interest (POI); illustrated for co-expression of GFP and RFP as POI1 and POI2, respectively. (b) Proposed mechanism of DI mediated autocleavage of a POI1-DI-POI2 polyprotein precursor. Arrows represent the general intein cleavage (pathways A & B).

The rationale behind development of the DI system is that N- and C-terminal autocleavage reactions conferred by engineered inteins can be isolated and tuned separately^19^. Here, each intein domain in the DI cassette is optimized independently, to maximize the N-terminal and C-terminal autocleavage activity of *Ssp* DnaE and DnaB intein, respectively. As shown in Fig. 1b, for the *Ssp* DnaE mini-intein moiety of the DI system (**1**), we created the Ala mutation at position 159 (N159A) to abolish Asn cyclization, and incorporated an Asp residue at position −1 (Asp-1) that has been reported to accelerate *Ssp* DnaE autocleavage at its N-terminal splicing junction^20^. With the N159A mutation, release of the N-extein is believed to proceed via hydrolysis of the labile thioester bond in the linear (**2**) and branched (**3**) intermediates following the general N-terminal intein autocleavage (Fig. 1b, Pathways A & B)^19^. For the *Ssp* DnaB mini-intein moiety in **1**, we incorporated the Ala mutation at position 1 (C1A) that blocks the formation of thioester linkage preceding the N-terminus of intein. In addition, the Ser-Arg residues that were experimentally confirmed to give high C-terminal autocleavage activity, were incorporated after the C-terminal junction at position +1 and +2 of DnaB intein moiety, respectively^21^. Based on the engineered DI system, we assembled a panel of reporter polyprotein constructs to systematically investigate key factors governing the autoprocessing activity of the DI domain. Each of these constructs encodes a polyprotein (Fig. 1a) in which the DI sequence is sandwiched between an upstream GFP variant (GFP_172_; POI1)^22^, and a downstream mCherry sequence carrying a C-terminal Strep tag (RFP_Strep_; POI2). T7 promoter, human cytomegalovirus (CMV) promoter, and (ocs)_3_/mas promoter were used to drive expression of the DI-based polyproteins in *E. coli*, mammalian HEK293T cells and *Nicotiana tabacum*, respectively.

### Cellular processing of the dual-intein polyprotein in bacterial, mammalian and plant expression systems

**Figure 2:**
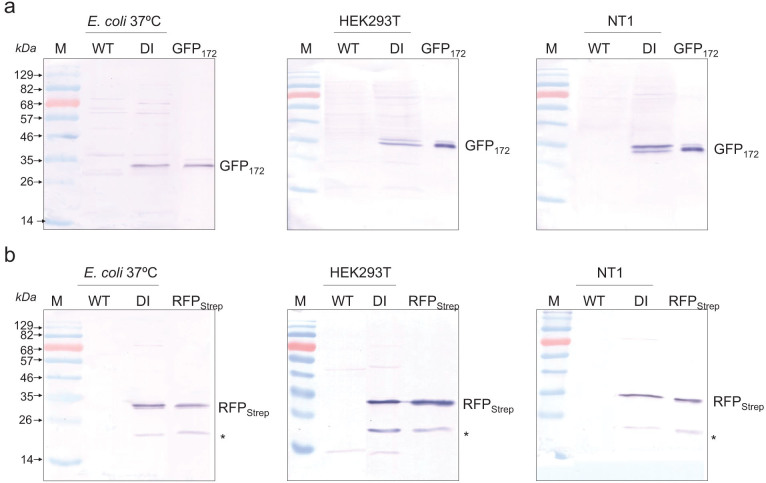
Dual-intein mediated autoprocessing of GFP_172_-DI-RFP_Strep_ polyprotein in *E. coli*, mammalian HEK293T, and tobacco NT1 cells, probed using western blots of soluble protein extracts. (a) Release of upstream protein probed using an anti-GFP antibody. (b) Release of downstream protein was confirmed using an anti-Strep tag antibody. Hereinafter, “M” represents protein markers, “WT” means non-transformed wild-type control, and “*” denotes a degraded RFP_Strep_ fragment resulting from sample preparation for gel electrophoresis (see text for details).

**Figure 3:**
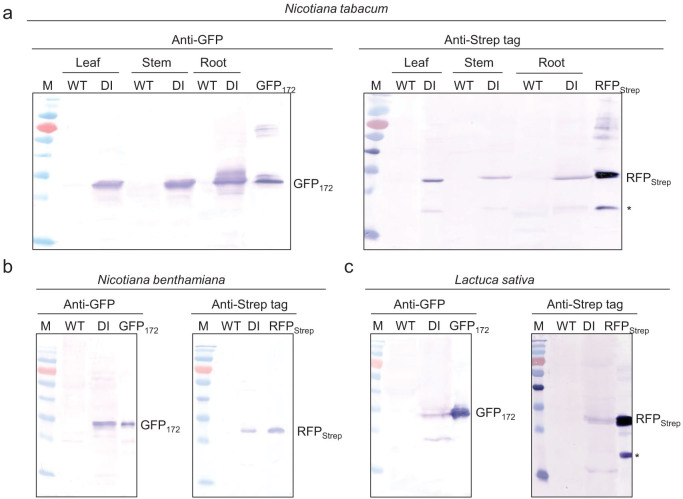
Processing of the GFP_172_-DI-RFP_Strep_ polyprotein in plants, probed with western blots. (a) Leaf, stem, and root tissues of stably transformed *N. tabacum* plants. (b) Leaf tissues of agroinfiltrated *N. benthamiana*. (c) Leaf tissues of agroinfiltrated *L. sativa L. var. longifolia*.

To investigate the extent of DI-mediated polyprotein autoprocessing, protein extracts from *E. coli*, HEK293T, and tobacco NT1 cells expressing the GFP_172_-DI-RFP_Strep_ reporter construct were subject to analysis of anti-GFP and anti-Strep tag western blots. As shown in Fig. 2, both GFP_172_ and RFP_Strep_ were nearly completely released from the polyprotein precursor in all tested host systems. The lower immuno-reactive band appeared in the anti-Strep tag western blot represents a partially hydrolyzed mCherry product that results from sample preparation for gel electrophoresis^23^. Processing of the DI polyprotein system at the whole plant level was investigated in transgenic *N. tabacum* cv. Xanthi plants expressing the GFP_172_-DI-RFP_Strep_ reporter construct. As with undifferentiated tobacco NT1 cells, efficient cleavage on both N- and C-termini of the DI fusion domain was observed in leaf, stem and root tissues based on the western blot analysis (Fig. 3a). While no significant difference in the extent of cleavage was noted between different plant tissues, highest expression level was observed in root tissue. This is due mainly to the (ocs)_3_/mas promoter used which gives highest expression in roots as reported in both tobacco and maize plants^24^,^25^. In addition to *N. tabacum*, efficient processing of the DI polyprotein was also seen in leaf tissues of *Nicotiana* *benthamiana* and *Lactuca sativa L. var. longifolia* (Romaine lettuce) by transient expression *via* agroinfiltration (Fig. 3b and 3c). Demonstration of the efficacy of the DI system in *N.* *benthamiana* is of particular interests as agroinfiltration of this plant species offers a very promising large scale recombinant antibody production platform which was used to produce ZMapp, a promising drug to fight the Ebola outbreak^26^,^27^.

### Determination of the cleavage sites in the dual-intein polyprotein precursor

**Figure 4:**
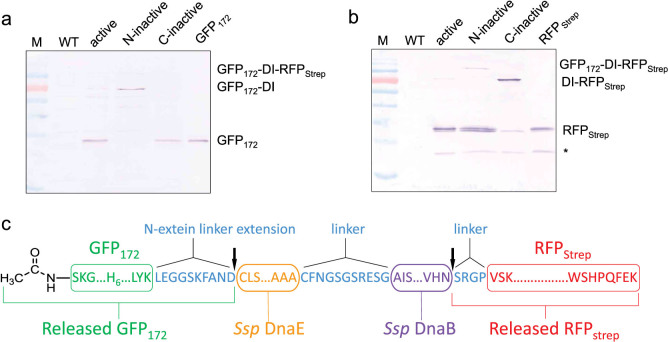
Characterizing autoprocessing of the GFP_172_-DI-RFP_Strep_ polyprotein. (a, b) Western blot analysis of cleavage patterns of polyproteins carrying active vs. N- or C- inactive DI variants confirmed release of the constituent proteins (GFP_172_ and RFP_Strep_) was attributed to the autocatalytic cleavage activity of the DI domain. The N- or C-terminal cleavage activity of the DI domain was blocked by Ala mutation at the respective cleaving junction to create N- and C-inactive DI mutant. (c) Organization of the polyprotein precursor showing the main protein domains (boxed) and the linker sequences (in blue); N-terminal amino acid sequencing and ESI-TOFMS analysis of the released proteins revealed cleavage of the polyprotein at the expected sites indicated by the arrows.

To further investigate whether the observed cellular cleavage of the precursor polyprotein was specifically attributed to the autocatalytic cleavage activity of the DI fusion domain, inactive intein mutants with blocked N- or C-terminal auto-cleavage activity were used to replace the cleavage-active inteins in the DI domain. Specifically, the first (Cys, position 1 of DnaE intein) or the last (Asn, position 154 of DnaB intein) residue in the DI domain was mutated to Ala to create an N-inactive or a C-inactive DI, respectively. As shown in Fig. 4a, when the N-terminal cleavage-inactive DI was used, the upstream GFP_172_ was unable to separate from the DI domain, yet since the C-terminal cleavage was still active, the downstream RFP_Strep_ could still be released from the polyprotein precursor. Similarly, for the C-terminal cleavage-inactive DI, the upstream GFP_172_ was liberated from the DI domain but the downstream RFP_Strep_ was not (Fig. 4b). This result confirmed that the highly efficient polyprotein auto-cleavage observed with the active DI domain was indeed resulted from the specific action of the DI domain as described by the intein cleavage mechanism (Fig. 1b).

The exact cleavage sites within the GFP_172_-DI-RFP_Strep_ polyprotein were determined by analyzing the released RFP_Strep_ and GFP_172_, purified from the transgenic tobacco NT1 cells. According to N-terminal sequencing, the RFP product released from the polyprotein contains an N-terminal Ser at position +1 of DnaB intein (followed by the Arg-Gly-Pro sequence), while the N-terminus of the released GFP_172_ was found to be blocked. As shown in Fig. 4c, the Ser-Arg-Gly-Pro sequence is flanking the C-terminal splicing junction of the DnaB intein, and hence it confirmed the release of the downstream RFP is mediated by the C-terminal auto-cleavage action of the intein. Furthermore, the observed molecular mass of the released GFP_172_ product measured using ESI-TOFMS is 28,590.90 Da which matches that of an N-terminal acetylated GFP_172_ (calculated mass 28,593.08 Da). As the most common substrate for N-terminal acetyltransferase, Ser at N-terminus of the upstream GFP_172_ was likely to be acetylated after the initiator Met was removed by methionine aminopeptidase. Taken together, these findings supported that the observed *in vivo* processing of the DI polyprotein precursor indeed occurred at the anticipated sites bordering the DI domain.

### Effects of cellular redox environment and culture temperature on the dual-intein autocleavage activity

**Figure 5:**
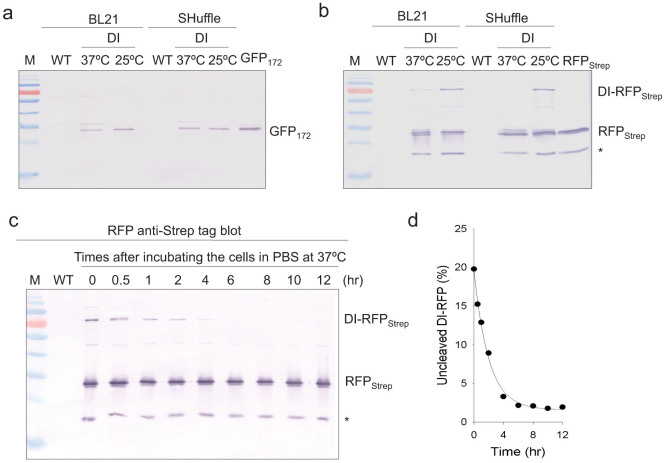
Effects of cellular redox environment and culture temperature on DI autocleavage activity. (a, b) Effect of cellular redox environment and expression temperature on the cleavage efficiency of the DI domain. (c) C-terminal cleavage efficiency of the DI domain in *E. coli* was improved by incubating at 37°C after overnight expression at 25°C. (d) Nearly complete cleavage was observed within 4 hours after incubation at 37°C.

Since kinetics of intein autocleavage could be affected by expression temperature and differential cellular redox environment, we investigated how these factors could affect the DI mediated polyprotein processing in *E. coli*. The GFP_172_-DI-RFP_Strep_ reporter construct was mobilized into *E. coli* BL21 and SHuffle^28^ strains engineered to have different cellular redox environments, and induced the protein expression at two different temperatures (25 vs. 37°C). Our results showed little difference in the polyprotein processing between the two strains tested (Fig. 5a and 5b). As shown in Fig. 2a and 2b, efficient polyprotein processing was also noted in HEK293T and tobacco NT1 cells which had a more oxidative cytosolic environment compared with *E. coli*. Therefore, with the highly efficient *Ssp* DnaE and DnaB mini-intein domains, self-excising activity of our DI system was not susceptible to differences in the host cell redox environment, at least within the differences found in the hosts tested.

As for the temperature effect, overnight induction of *E. coli* at 25°C did not affect the N-terminal cleavage efficiency of the DI domain (Fig. 5a). However, detection of the DI-RFP_Strep_ fragment on the western blot indicated less efficient C-terminal cleavage at 25°C, although the properly cleaved downstream RFP remained the predominant product based on the western blot analysis (Fig. 5b). Previous intein studies suggested that the N-terminal Cys or Ser residue at position 1 of intein played a crucial role in promoting Asn cyclization^29^. After trans(thio)esterification, the side chain of the liberated N-terminal Cys or Ser of intein nucleophilically attacked the scissile peptide bond at the C-terminal Asn, forming a transient macrocyclic intermediate that promotes Asn cyclization. When the N-terminal nucleophilic residue (position 1) was substituted with non-functioned Ala, reduction in C-terminal intein cleavage rate was observed^30^,^31^. In our DI design (Fig. 1b), C1A mutation was introduced into the C-terminal cleaving intein (*Ssp* DnaB in **1**) to enforce the intein reaction towards C-terminal cleavage, thus the effect of the first nucleophilic side chain was eliminated. To this end, the Asn cyclization was mainly initiated by protonation of the imidazole side chain of the penultimate His (position 153 of DnaB intein)^32^,^33^. The attenuated C-terminal cleavage rate of the C1A substitution could be compensated by increased hydrolysis rate at elevated reaction temperature. To this end, after overnight induction at 25°C, the *E. coli* cells were resuspended in PBS buffer and incubated at 37°C for up to 12 hours. Nearly complete release of RFP_Strep_ from the DI-RFP_Strep_ fragment was observed after about only four hours (Fig. 5c and 5d). This post-induction incubation strategy provides a solution to remedy the effect of the attenuated C-terminal cleavage at lower temperature. Since only the *Ssp* DnaB intein was tested in this study as the C-terminal cleavage domain, this intein might be replaced by another intein capable of more active C-terminal cleavage. Such intein variants have been reported, for instance by Ramirez *et al*^34^.

### Characterization of proteins released from the dual-intein polyprotein precursor

**Figure 6:**
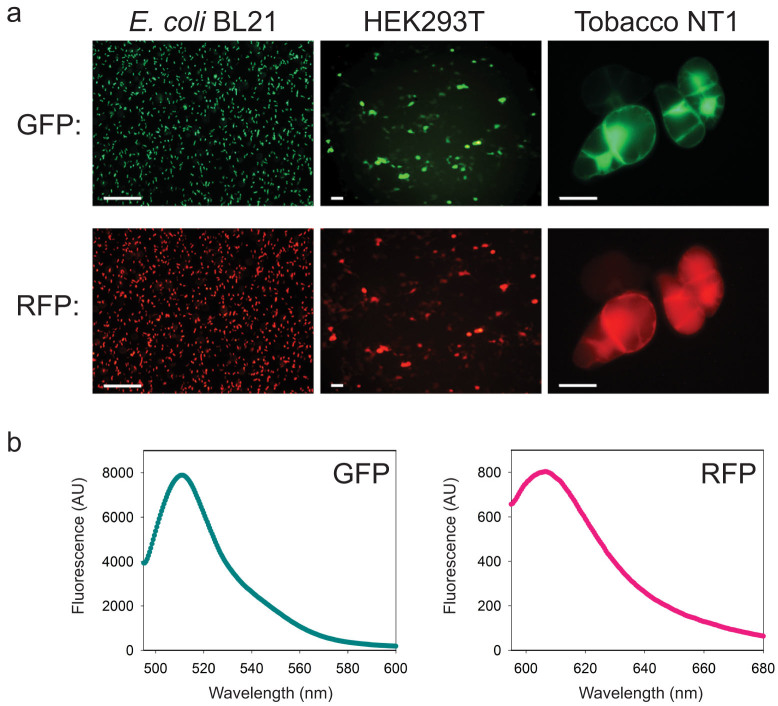
Processed POIs from the DI-based polyprotein precursor preserve proper function. (a) Cells expressing the GFP_172_-DI-RFP_Strep_ reporter construct display both green (upper panel) and red (lower panel) fluorescence. Scale bar: 50 μm. (b) GFP_172_ and RFP_Strep_ released from the polyprotein precursor are functional as indicated by their respective fluorescence emission spectra.

To determine whether proteins of interest released from the polyprotein precursor remain functional, transgenic cells expressing the GFP_172_-DI-RFP_Strep_ reporter were examined using fluorescence microscopy. As shown in Fig. 6a, bright green and red fluorescence distinctive from the background autofluorescence were visible in all tested cell lines. These results, in consort with the western blot data (Fig. 2) that showed essentially complete cleavage of the polyprotein precursor, indicated that fluorescence detected in the transgenic cells was originated from the processed proteins and hence these proteins are indeed functional. As a side note, slightly different distribution of GFP and RFP can be seen in the plant cell images in Fig. 6a. Unlike the cytoplasmic distribution of RFP fluorescence, GFP was prone to accumulation in the nuclear region of tobacco cells although no nuclear localization signal had been identified in its sequence^35^,^36^. Nuclear localization of GFP was thought to be resulted from the passive diffusion through the nuclear pores due to their low molecular mass^36^,^37^. Once inside the nucleus, GFP oligomerized into large homomultimers for the high local concentration, trapping the GFP in the nuclear boundary and leading the equilibrium towards nucleus import^36^. On the other hand, RFP (mCherry) tends to maintain its monomeric structure and traverse freely through the nuclear pores. Further analysis of the transgenic cell extracts using fluorescence spectrometry showed emission spectra distinctive to GFP and RFP (mCherry), indicating that not only the released GFP and RFP displayed fluorescence, the spectral properties of the released fluorescent proteins were not affected by the DI-mediated *in vivo* cleavage (Fig. 6b).

**Figure 7:**
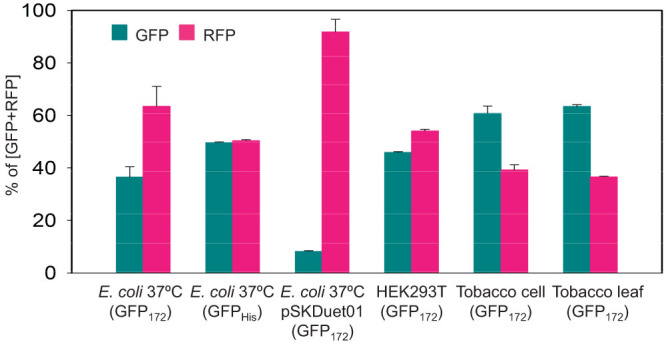
Relative expression levels of the constituent proteins released from the GFP-DI-RFP polyprotein precursor in different expression hosts.

In multi-protein co-expression, it is important to be able to control the relative stoichiometry of the gene products. To determine whether stoichiometric accumulation of the constituent proteins can be achieved using the DI polyprotein approach, cellular accumulation of cleaved GFP and RFP was analyzed using scanning densitometry of western blots, along with protein quantification based on fluorescence measurement. As shown in Fig. 7, approximately equal molar accumulations of processed GFP_172_ and RFP_Strep_ were noted in mammalian HEK293T cells. However, production of GFP_172_ was about 50% lower than RFP_Strep_ in *E. coli* BL21 induced at 37°C. The GFP_172_ variant contained an internal hexa-histidine sequence inserted between GFP residues 172 and 173, and it was found in our earlier study to fold less efficiently in *E. coli* at 37°C than at 25°C.^22^ When GFP with a C-terminal His tag (GFPHis) that folds well at 37°C replaced GFP_172_ in the polyprotein vector, it resulted in nearly equimolar accumulation of GFP_His_ and RFP_Strep_ (Fig. 7). This result illustrated the fact that post-translational modification and/or differential protein stability could also play a role in deciding the relative accumulation levels of the constituent proteins released from the polyprotein. For proteins with similar stability or folding efficiency, we showed here that stoichiometric production could indeed be achievable in *E. coli* with the DI polyprotein vector system when the GFP_His_ was used to replace the upstream GFP_172_. Such stoichiometric expression is an important feature that makes the DI polyprotein system superior to expression vectors containing multiple monocistronic expression cassettes. To this end, we observed that GFP_172_ and RFP_Strep_ protein levels differed by over 10-fold when co-expressed using the pSKDuet01 (Addgene plasmid 12172) vector which is derived from pRSFDuet-1^8^ that contains two expression cassettes (Fig. 7).

In mammalian HEK293T cells, GFP_172_ apparently folded relatively efficiently at 37°C, reaching almost similar expression levels as RFP_Strep_ (Fig. 7). In plant hosts, for both undifferentiated cultured tobacco cells and leaf tissue of transgenic tobacco, the downstream RFP_Strep_ accumulated to a lesser amount than GFP_172_. As discussed above, decline in C-terminal intein cleavage was observed in *E. coli* cells cultured at lower temperature (e.g. at 25°C). This attenuated cleavage efficiency was likely caused by the intrinsic C1A mutation in *Ssp* DnaB mini-intein domain that reduced reaction rate of C-terminal Asn cyclization in a temperature-dependent manner. Thus, similar reduction in C-terminal cleavage could also be expected in plants that preferably grown at room temperature. Failure in detection of the uncleaved DI-RFP_Strep_ fragment in plant extracts by western blot suggested its degradation inside the cells.

**Figure 8:**
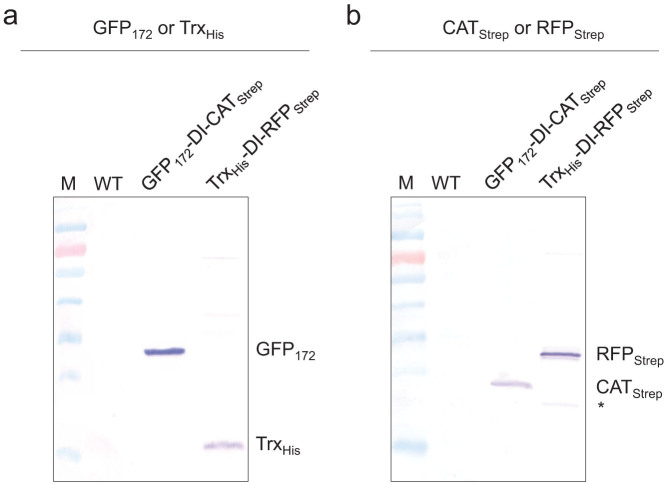
DI polyprotein system is also applicable to co-expressing proteins other than fluorescent reporters. Efficient release of protein constituents from GFP_172_-DI-CAT_Strep_ and Trx_His6_-DI-RFP_Strep_ polyproteins was detected using (a) anti-His tag, and (b) anti-Strep tag western blots.

To further demonstrate the general applicability of the DI polyprotein vector system for multi-protein co-expression, upstream GFP_172_ and downstream RFP_Strep_ were replaced by a C-terminal His tagged thioredoxin (Trx_His_) and a C-terminal Strep tagged chloramphenicol acetyltransferase (CAT_Strep_), respectively, to create constructs Trx_His_-DI-RFP_Strep_ and GFP_172_-DI-CAT_Strep_. As revealed by the anti-His tag and anti-Strep tag western blots, highly efficient processing of the polyprotein precursors with essentially complete release of the constituent proteins in *E. coli* BL21 was achieved in both cases (Fig. 8). The bioactivity of the released CAT enzyme was also confirmed by enzymatic assay. Based on scanning densitometry analysis of western blots, Trx_His_ and RFP_Strep_ were found to accumulate to approximately equimolar amounts in *E. coli* cells expressing Trx_His_-DI-RFP_Strep_. As for the GFP_172_-DI-CAT_Strep_ vector, GFP_172_ accumulated to a lesser amount than CAT_Strep_, which again was most likely attributed to the less efficient folding of GFP_172_ in *E. coli* BL21 at 37°C, as discussed above. Nonetheless, when *E. coli* transformed with the GFP_172_-DI-CAT_Strep_ vector was induced overnight at 25°C, the molar ratio between GFP_172_ and CAT_Strep_ became very close to one. These results point to potential universal applicability of the DI-polyprotein vector system for multi-protein co-expression, as the autocleavage activity of the DI fusion domain is apparently quite insensitive to the flanking protein sequences.

### Protein expression levels using the dual-intein polyprotein vectors

Amounts of GFP_172_ generated using the DI-polyprotein vector (encoding GFP_172_-DI-RFP_Strep_) were compared with those using vectors harboring either a single non-fused GFP_172_ or separate GFP_172_ and RFP_Strep_ expression cassettes, under the control of the same promoter. This comparison was carried out in *E. coli* and tobacco NT1 cells, using the T7 and the (ocs)_3_/mas promoter, respectively. We investigated *E. coli* BL21(DE3) and the SHuffle^28^ strain for GFP_172_ expression from the DI polyprotein vector vs. the pSKDuet vector (co-expressing GFP_172_ and RFP_Strep_ from separate monocistronic cassettes on the same vector). The SHuffle strain constitutively expresses DsbC which is a disulfide bond isomerase known to serve as a chaperone that assists in protein folding^38^. Interestingly, while GFP_172_ expression is lower when expressed using the DI vector than using pSKDuet in BL21(DE3) at 37°C, the trend is reversed at 25°C in both BL21 and SHuffle. At 37°C in the SHuffle strain, GFP_172_ expression is similar using either the DI vector or pSKDuet (Supplementary Fig. S2). Chaperone co-expression has been reported to cause proteolytic degradation of folding-reluctant protein species^39^, which might explain the low expression levels seen in the SHuffle strain at 37°C, as GFP_172_ is known to fold less efficiently than the regular GFP at this temperature^22^. The lower expression temperature (25°C) facilitated folding of GFP_172_ and the DI domain in both strains.

In tobacco, GFP_172_ reached 0.28% and 0.04% of total soluble proteins (TSP) when expressed from the GFP_172_-DI-RFP_Strep_ polyprotein vector in NT1 cells and *N. tabacum* leaf tissue, respectively. For comparison, expression of GFP_172_ or RFP_Strep_ alone using the same (ocs)_3_/mas promoter in transgenic tobacco NT1 cells reached about 0.29% and 0.61% TSP, respectively. Although more proteins need to be tested, the encouraging reporter protein expression results suggest that the DI-based polyprotein vector system could produce a comparable level of recombinant proteins as with the conventional single protein vectors.

### Autoprocessing activity of the DI domain without its N-terminal extein linker extension

In the data presented above, the DI domain employed contains an N-terminal extension derived from the native flanking N-extein sequence of the *Ssp* DnaE intein but with mutations at N-1 and N-2 positions to Asp and Asn, respectively (Fig. 4c). This linker extension was incorporated with the intention to accelerate autocleavage at the N-terminal of the DI domain^20^. While this version of the DI domain mediated highly efficient autocleavage as presented in the preceding sections, we have subsequently confirmed that comparable autocleavage can be attained by removing the DI N-terminal extension. As shown in the western blot result presented in Supplementary Fig. S3, co-expression of GFP_172_ and RFP_Strep_ using a polyprotein vector containing a modified DI domain omitting its N-terminal extension sequence, i.e. LEGGSKFAND (cf. Fig. 4c), led to very efficient release of both GFP_172_ and RFP_Strep_. This result is rational since it is known that the key residues involved in N-terminal intein autocleavage reside inside and immediately downstream of the intein sequence^19^. In this case, no extraneous amino acids are appended to the POI released from the N-terminal of the DI domain. As for the downstream POI, after it is released from the polyprotein precursor, and upon purification, the amino acid overhang at its N-terminus can be removed to preserve its authentic N-terminal residue using a suitable amino-peptidase. As an example, commercially available TAGZyme system based on the use of dipeptidyl aminopeptidase I (DAPase Enzyme) in combination with glutamine cyclotransferase (Qcyclase Enzyme) and pyroglutamyl aminopeptidase (pGAPase Enzyme) (Qiagen) may be considered. To this end, a DAPase stop point (e.g. a Gln residue) needs to be introduced in front of the native N-terminal residue. Upon removal of the first N-terminal dipeptide, DAPase removes dipeptides progressively till it encounters the Gln stop point which is then removed by Qcyclase and pGAPase^40^.

### Autoprocessing of polyprotein containing a single intein domain

In principle, self cleavage on both ends of an intein may be achievable by promoting hydrolysis of the linear (thio)ester intermediate and allowing more efficient intein C-terminal Asn cyclization^30^. It has been suggested that release of both N- and C-exteins from a single intein without subsequent extein splicing could occur by mutating the essential residue at position +1 (flanking the intein C-terminus) to Gly or Ala^30^. Here we investigated whether co-cleavage at both splicing junctions of a single intein with such mutation can result in efficient release of flanking POIs as in the case of DI-based vectors. A polyprotein construct that encodes an *Ssp* DnaE mini-intein with C + 1A mutation sandwiched between a GFP_172_ and a mCherry was created and expressed in *E. coli* BL21(DE3). Cell extract was probed with western blot. As shown in Supplementary Fig. S4, a substantial portion of uncleaved or partially cleaved polyprotein fragments was detected on western blots of the *E. coli* extract, while only a small portion (about 10%) resulted from co-cleavage at both ends of the intein. By comparing this result with that shown in Supplementary Fig. S3 for a DI-based polyprotein system, it is clear that the DI domain is necessary to confer hyperactive autocleavage of the polyprotein precursor to achieve highly efficient release of the POIs.

### Advantages and limitations of the DI polyprotein system

In this study we demonstrated that incorporation of an engineered DI domain in a polyprotein system enabled highly efficient synchronized protein co-expression from a single ORF in different expression host systems. This approach has a number of unique advantages over existing methods. The DI system is based on sequences of non-viral origins and hence avoids biosafety, environmental, and compliance issues. The highly efficient and rapid *in vivo* protein processing is autocatalytic and it does not require host-specific factors. Since the multiple POIs are encoded by a single ORF, it potentially allows control over the relative stoichiometry of the gene products to achieve a balanced multi-protein synthesis, especially when the POIs have similar folding and stability characteristics. The DI technology is applicable to both prokaryotic as well as eukaryotic hosts. By integrating with homologous recombination-based cloning methods such as the popular Gateway technology, the new DI polyprotein cassette can be easily shuttled between different hosts for expression. On the downside, with the DI based polyprotein vector the multiple POIs are expressed from the same promoter and thus if expression of each POI needs to be driven by a different promoter, e.g. for expression in different tissues or be expressed at different times, the DI polyprotein vector will not be well suited. Overall, the DI based polyprotein approach has many unique features and is also highly complementary to existing protein expression techniques, and hence it is a highly valuable addition to the molecular toolbox for synchronized multi-protein co-expression.

## Methods

### Vector construction

The Dual-intein (DI) polyprotein cassettes were cloned into different vectors for expression in *E. coli*, mammalian HEK293T, and tobacco NT1 cells, respectively. To assemble the GFP_172_-DI-RFP_Strep_ cassette, first the coding sequence of *Ssp* DnaE mini-intein (DnaE) N159A variant flanked by five bordering N-extein residues (KFAND) reported to accelerate N-terminal cleavage^20^ and three native C-extein (CFN) residues was synthesized by Genscript (Piscataway, NJ) and ligated into pUC57 between *Xho*I and *Xba*I sites to yield pUC-DnaE(A_159_CFN) vector. The coding sequence for GFP_172_ (a modified mGFP5 with six histidine residues inserted between amino acid residues 172 and 173)^22^ was amplified from pGEM5z-GFP172 incorporating *Sal*I and *Xho*I sites by forward (*Sal*I-mGFP5) and reverse primers (GFP-*Xho*I-R), respectively. mCherry coding region was amplified from pETMD^41^ to incorporate a 5′- *ApaI* site and a Strep tag followed by a *Sac*I site at the 3′-end by two step overlap PCR with the forward primer (mCherry-2A-F1) and reverse primers (mCherry-Strep and GKZ-R). C-terminal cleaving *Ssp* DnaB mini-intein (DnaB) with three native bordering N-extein residues (ESG) and C-terminal cleavage accelerating C-extein residues (SR)^21^ was amplified from pTWIN1 (New England Biolab, Ipswich, MA) with primer pairs (DnaB-*XbaI*-F/DnaB-SRGP-R) incorporating *Xba*I and *Apa*I flanking the 5′- and 3′- junctions, respectively. PCR fragments of GFP_172_, DnaB mutant and mCherry-Strep tag were successively ligated into pUC57-DnaE backbone after digestion with respective restriction enzymes to yield pUC-GFP_172_-DnaE-DnaB-mCherry-strep (called pUC-GFP_172_-DI-RFP_Strep_ hereinafter). To replace GFP_172_ with GFP_His_ (mGFP5 with C-terminal hexa His tag) in pUC-GFP_172_-DI-RFP_Strep_, GFP_His_ coding sequence was amplified from pBISN1-mGFP5-ER using primers GFP-*KpnI*-F and mGFP5-His-*XhoI*-R to incorporate *KpnI* and *XhoI* sites at the 5′- and 3′-ends, respectively, and replaced the *KpnI*-GFP_172_-*XhoI* fragment in pUC-GFP_172_-DI-RFP_Strep_ to yield pUC-GFP_His_-DI-RFP_Strep_. To assemble a vector for expressing thioredoxin-DI-mCherry (Trx_His_-DI-RFP_Strep_) in *E. coli*, the DI-RFP_Strep_ fragment from pUC-GFP_172_-DI-RFP_Strep_ was amplified using PCR primers *Kpn*I-DnaE-F and GKZ-R to incorporate *Kpn*I and *Sac*I sites at the 5′- and 3′-ends, respectively. The PCR fragment was digested with *Kpn*I/*Sac*I and ligated into *Kpn*I/*Sac*I digested pET32a vector for in-frame fusion with an upstream thioredoxin sequence to create pET-Trx_His_-DI-RFP_Strep_.

For protein expression, the assembled GFP_172_-DI-RFP_Strep_ cassette was mobilized into pET vector. In doing so, *Nde*I site within the GFP_172_ coding region was removed by silent mutation using overlap PCR with primers *Nde*I-G172-F/Nd172R and Nd172F/G172-*Kpn*I-R. The modified GFP_172_ incorporating a 5′-*Nde*I and a 3′-*Xho*I site was subsequently cloned into pET-Trx_His_-DI-RFP_Strep_ vector to replace the upstream thioredoxin, yielding pET-GFP_172_-DI-RFP_Strep_. To replace the downstream RFP_Strep_ protein in DI polyprotein to CAT_Strep_, CAT_Strep_ coding sequence was amplified from pET-CAT vector by two step PCR using forward primer (CAT-2A-F1) and reverse primers (CAT-STREP-R and GKZ-R) incorporating *Apa*I and *Sac*I at 5′- and 3′- end, respectively, and replaced the *Apa*I-mCherry-streptag-*Sac*I fragment in pET-GFP_172_-DI-RFP_Strep_ to create pET-GFP_172_-DI-CAT_Strep_. To eliminate the C-terminal overhang on the upstream POI, the N-extein linker extension sequence (LEGGSKFAND) preceding the DI domain in the GFP_172_-DI-RFP_Strep_ polyprotein was removed by mutating the pET-GFP_172_-DI-RFP_Strep_ vector via inverse PCR using primer pair G172-DnaE-F and G172-DnaE-R. The amplified plasmid pET-GFP_172_-DI(−)-RFP_Strep_ was transformed into *E. coli* DH5α after *Dpn*I enzyme treatment. To compare protein expression using our DI polyprotein system with the commercial vector incorporating multiple monocistronic expression cassettes, GFP_172_ and RFP_Strep_ were inserted into the first and the second multiple cloning sites of the pSKDuet01 vector (Addgene plasmid 12172), respectively. To do so, RFP_Strep_ fragment was PCR amplified from pETMD vector using forward primer (XBF) and reverse primer (MBR) containing a 5′-*Nde*I and a 3′-*Bam*HI, respectively. Amplified *Nde*I-RFP_Strep_-*Bam*HI fragment was ligated into pSKDuet01 vector digested with the corresponding restriction enzymes, to yield pSKM. GFP172 incorporating a *Sac*II site and a *Sac*I site at the 5′- and 3′-ends, respectively, was amplified from pGEM5z-GFP172 vector using primers GFS2 and GRS. The resulting *Sac*II-GFP_172_-*Sac*I fragment was ligated into pSKM vector digested with *Sac*II and *Sac*I enzymes to yield pSKGM. To compare the co-cleavage efficiency at both N- and C-termini of our DI fusion domain with the single intein incorporating the C + 1A mutation, *Xho*I-Ssp DnaE(A_159_CFN)-*Xba*I fragment in pUC-DnaE(A_159_CFN) vector was replaced with *Xho*I-Ssp DnaE(N_159_AFN)-*Xba*I which was amplified using primers *Xho*I-Int-F and DnaENAFN-*Xba*I-R, to yield pUC-DnaE(N_159_AFN). The resulting *Xho*I-Ssp DnaE((N_159_AFN)-*Xba*I fragment along with a C-terminal linker sequence was excised from pUC-DnaE(N_159_AFN) vector between *Xho*I and *Apa*I sites and was subsequently used to replace the DI fragment in pUC-GFP_172_-DI-RFP_Strep_ vector, to yield pUC-GFP_172_-Int-RFP_Strep_.

The cleavage-inactive DnaE intein mutant was generated using primers (IDnaE-*Kpn*I-F/DnaE-*Xba*I-R-2) that carry the C1A mutation (in addition to the N159A mutation). This inactive DnaE replaced its active counterpart in pET-GFP_172_-DI-RFP_Strep_ to create pET-GFP_172_-DI(N-)-RFP_Strep_ that encodes an N-(cleavage) inactive DI polyprotein. Here, the N-extein and C-extein residues flanking the inactive DnaE were changed to EY and GG, respectively, to minimize any cleaving activity. Similarly, cleavage-inactive DnaB intein mutant was generated using primers (DnaB-*Xba*I-F/IDnaB-*Apa*I-R) that contains N154A mutation (in addition to the C1A mutation). In addition, C-extein residues bordering DnaB were changed from SR to GS. The inactive DnaB fragment was digested with *Xba*I/*Apa*I, and ligated into the pET-GFP_172_-DI-RFP_Strep_ vector digested with the corresponding restriction enzymes, yielding a vector pET-GFP_172_-DI(C-)-RFP_Strep_ that encodes a C-(cleavage) inactive DI polyprotein.

For tobacco NT1 cell transformation, coding sequence of GFP_172_-DI-RFP_Strep_ was inserted between *Sal*I and *Sac*I sites in the binary vector pE1775 under the control of the mannopine/octopine synthase (ocs)_3_/mas promoter^25^. For mammalian HEK293T transient expression, GFP_172_-DI-RFP_Strep_ fragment was PCR amplified using primers *Sac*I-*Nhe*-DI-F3 & Strep-*Not*I-R to incorporate *Sac*I and *Not*I at 5′- and 3′- ends, respectively, and inserted into pcDNA3.1 vector at the corresponding digested sites.

### Expression in *Escherichia coli*

The pET vectors encoding DI-polyprotein sequences were transformed into *E. coli* BL21(DE3). Bacterial cells were cultured at 37°C except when indicated otherwise. Protein expression was induced with 0.1 mM of isopropyl-β-D-thiogalactopyranoside (IPTG) when cells were grown to an OD_600_ of 0.5. In addition to *E. coli* BL21(DE3), protein expression was performed in SHuffle *E. coli* strain (New England Biolab, Ipswich, MA) lacking the trxB and gor reductases along with an additional suppressor mutation (ahpC) which restores viability^28^. These mutations lead to an altered redox state in the SHuffle cells to permit oxidative folding. Upon completion of induction, cells were pelleted by centrifuging at 6000 × g for 5 min, and then rinsed using phosphate buffer saline for three times. Washed cell pellets were disrupted by ultrasonication at 5 Watt for 5 min in PBS protein extraction buffer (20 mM sodium phosphate pH 7.5, 150 mM sodium chloride, 1 mM phenylmethylsulfonyl fluoride, 200 μM lysozyme, 1 μg/ml leupeptin, 100 ng/ml pepstatin). Total soluble proteins were obtained by centrifuging for 15 min at 14,000 × g at 4°C and used in subsequent analysis.

### Mammalian HEK293T cell transfection, protein expression, extraction, and analysis

DI mediated polyprotein processing in mammalian system was tested in human embryonic kidney (HEK293T) cells. HEK293T cells were cultured in DMEM medium (Invitrogen, Grand Island, NY) supplemented with 10% fetal bovine serum (FBS) and 50 mg/L penicillin-streptomycin, and incubated at 37°C under 5% CO_2_. Transient transfection was performed using Xtreme Gene 9 (Roche, Indianapolis, IN) when the cell density reached 70–90% confluency, by following the product instruction. The transfection complex was subsequently transferred to 3 ml of HEK293T culture. After 24 hour of incubation at 37°C under 5% CO_2_, transfected cells were washed and resuspended in PBS protein extraction buffer, and then lysed by ultrasonication at 5 watt on the ice for 5 min to extract the total soluble protein. Protein extract was clarified by centrifugation at 14,000 × g at 4°C for 15 min and subjected to subsequent analysis.

### Tobacco transformation and protein characterization

The GFP_172_-DI-RFP_Strep_ polyprotein binary vector was transformed into *Agrobacterium tumefaciens* C58C1 by electroporation. After PCR confirmation, transformed *Agrobacteria* was co-cultivated with the tobacco NT1 cells or tobacco leaves (*N. tabacum* cv Xanthi) using a modified protocol reported previously^42^,^43^. The resultant transformants were selected on MS agar media^44^ supplemented with hygromycin. The high expressing lines, screened based on GFP fluorescence and western blot analysis, were selected for subsequent characterization. Transient expression was performed in leaf tissues of *N. benthamiana* and Romaine lettuce via vacuum assisted agroinfiltration as described in previous publication^45^. Protein extraction from transgenic tobacco was carried out by following a previously published protocol^46^. Clarified supernatants were used for subsequent western blot, protein, fluorescence analysis, and chromatographic purification. Fluorescence measurement was performed using a Hitachi F-2500 fluorescence spectrophotometer with excitation at 470 and 575 nm for detection of GFP and mCherry fluorescence, respectively. Amount of GFP and mCherry was estimated based on the fluorescence calibration curve established using purified protein standards.

### Purification of cleaved GFP_172_ for ESI-TOFMS analysis

Crude protein extracts from transgenic tobacco NT1 calli were pre-clarified by 30% ammonium sulfate precipitation before applying onto a Phenyl-Sepharose hydrophobic-interaction column (GE Healthcare, Piscataway, NJ) connected to a Biologic Duo Flow chromatography system (Bio-Rad, Hercules, CA). The loaded column was rinsed with a washing buffer (20 mM sodium phosphate pH 8.0, 1 M ammonium sulfate), and eluted with a linear gradient from 1 M to 0 M ammonium sulfate over 20 column volumes. Fractions with GFP fluorescence were subsequently purified with a HiTrap IMAC-Sepharose column (GE Healthcare, Piscataway, NJ) by washing with a binding buffer (20 mM sodium phosphate pH 7.4, 500 mM sodium chloride and 20 mM imidazole) and eluted with a linear gradient from 20 mM to 500 mM imidazole over 12 column volumes. Fractions containing GFP fluorescence were concentrated and desalted with a Zeba desalting spin column (Thermo Fisher., Rockford, IL) for electrospray time-of-flight mass spectrometry (ESI-TOFMS) analysis on an Agilent 6210 LC-TOFMS system fitted with an ESI source operated in positive ion mode as described previously^45^.

### Purification of cleaved RFP_Strep_

Cleaved RFP_Strep_ was purified with a Strep-Tactin Superflow Plus 2 ml column according to the manufacture manual (Qiagen, Valencia, CA). Briefly, the column was charged with crude soluble protein extracts from transgenic tobacco NT1 cells then washed with NP buffer (50 mM sodium phosphate pH 8.0, 300 mM sodium chloride), followed by elution with NPD buffer (NP buffer containing 2.5 mM desthiobiotin). The fractions shown mCherry fluorescence were concentrated for N-terminal amino acid sequencing.

### N-terminal amino acid sequencing

The GFP_172_ and RFP_Strep_ liberated from the polyprotein precursor were purified by chromatography as described above, followed by separation on SDS-PAGE and transferred onto a PVDF membrane. The target band with the expected size was excised out after staining with Coomassie blue. The N-terminal sequencing was performed using Edman degradation on a Perkin Elmer Applied Biosystems Procise 494 protein/peptide sequencer coupled with an on-line Perkin Elmer Applied Biosystems Model 140C PTH Amino Acid Analyzer (Applied Biosystems, Calsbad, CA), performed by the Protein Core Facility at the Iowa State University.

### Additional methods

Detailed methodology of gel electrophoresis, western blot analysis, and CAT assay, along with a list of primers used in cloning can be found in the Supplementary Information.

## Author Contributions

B.Z. designed and conducted experiments, analyzed data, and assisted in writing the paper. M.R. conducted initial cloning and expression experiments. Z.H. assisted in cloning and protein expression. Z.L. and P.W. assisted in ESI-TOFMS analysis and editing the paper. W.S. conceived the research, designed experiments, analyzed data, and wrote the paper.

## Supplementary Material

Supplementary Information Supplementary Information
