# Supplementary material for: A Dual-Intein Autoprocessing Domain that Directs Synchronized Protein Co-Expression in Both Prokaryotes and Eukaryotes
Source: Sci Rep. 2015 Feb 25;5:8541. doi: 10.1038/srep08541 (PMC4339811; doi:10.1038/srep08541)
Supplement: Supplementary Information [file srep08541-s1.pdf]

Supplementary Information

A Dual-Intein Autoprocessing Domain that Directs Synchronized Protein Co-Expression in Both Prokaryotes and Eukaryotes

Bei Zhang[†], Madhusudhan Rapolu[†], Zhibin Liang[‡], Zhenlin Han[†], Philip Williams[‡], Wei Wen Su[*†]

[†]Department of Molecular Biosciences & Bioengineering, University of Hawaii at Manoa, Honolulu, Hawaii 96822, USA

[‡]Department of Chemistry, University of Hawaii at Manoa, Honolulu, Hawaii 96822, USA

*To whom correspondence should be addressed: email: wsu@hawaii.edu. Tel: 1(808)956-3531. Fax: 1(808)956-3542

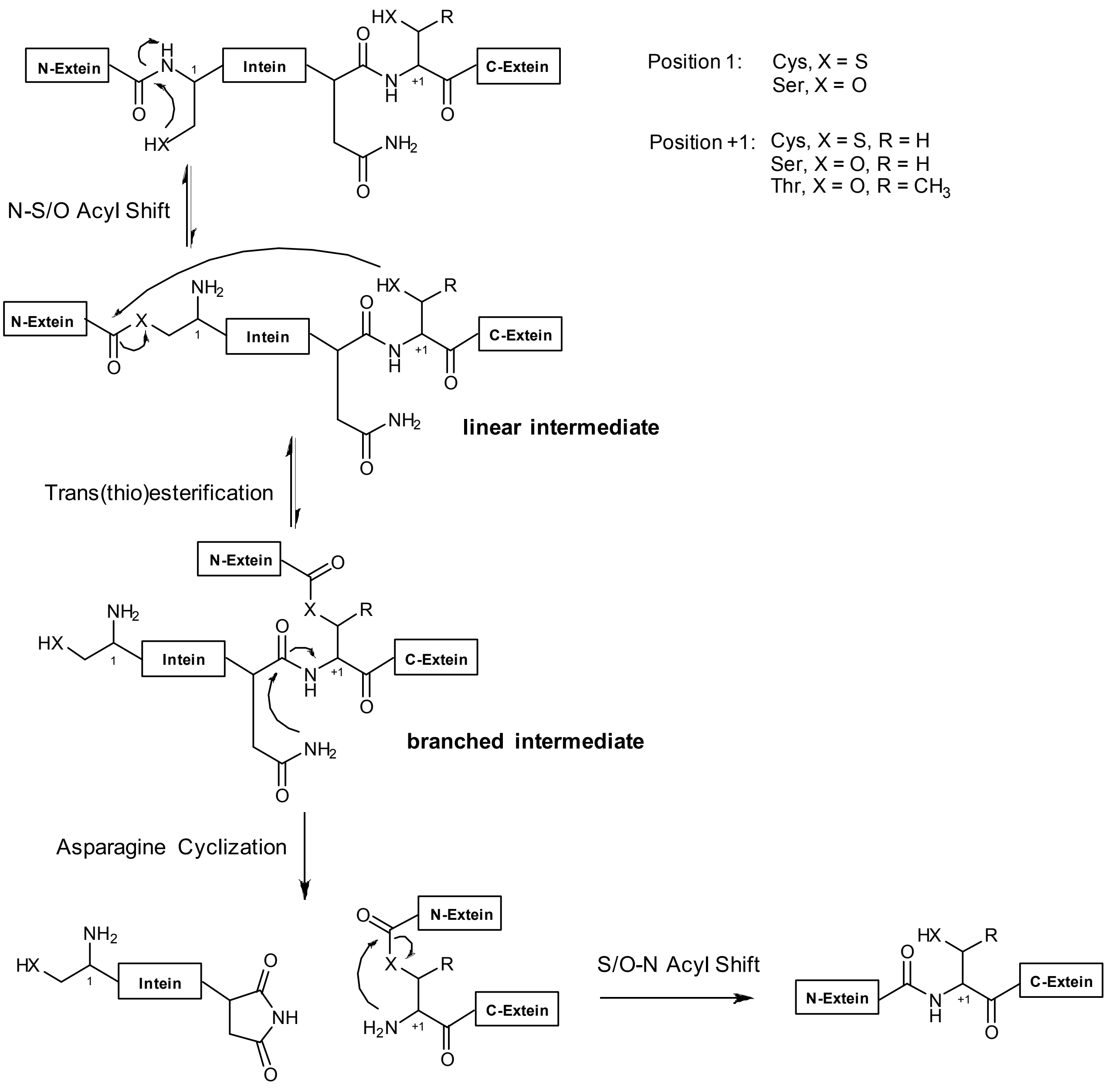

Supplementary Figure S1 | Mechanism of intein mediated protein splicing.

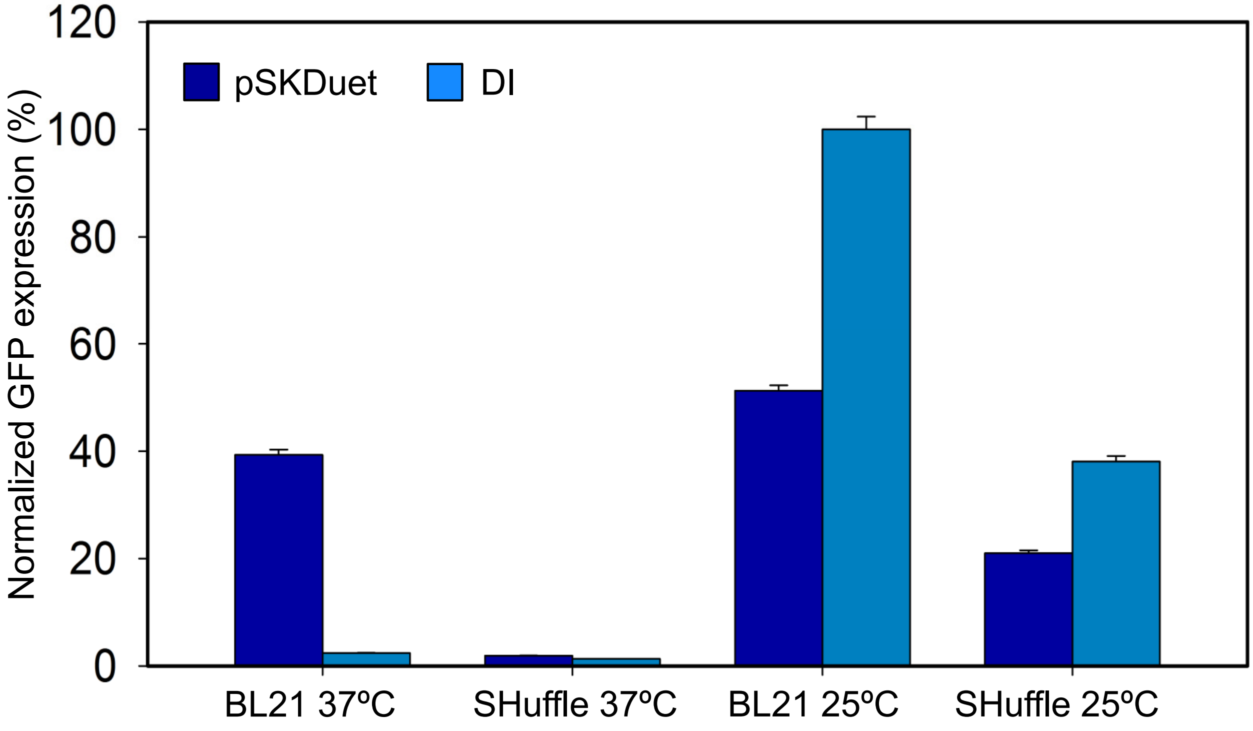

Supplementary Figure S2 | Comparison of protein expression levels using the DI based polyprotein vector vs. the duet vector in *E. coli* BL21(DE3) or SHuffle strain under the control of T7 promoter. Protein expression was induced by 0.1 mM IPTG at 37°C vs. 25°C. Protein level after overnight expression was determined based on the percentage of GFP_{172} in total soluble proteins, and normalized by considering the highest expression sample (i.e. expression with DI in BL21 at 25°C) as 100%.

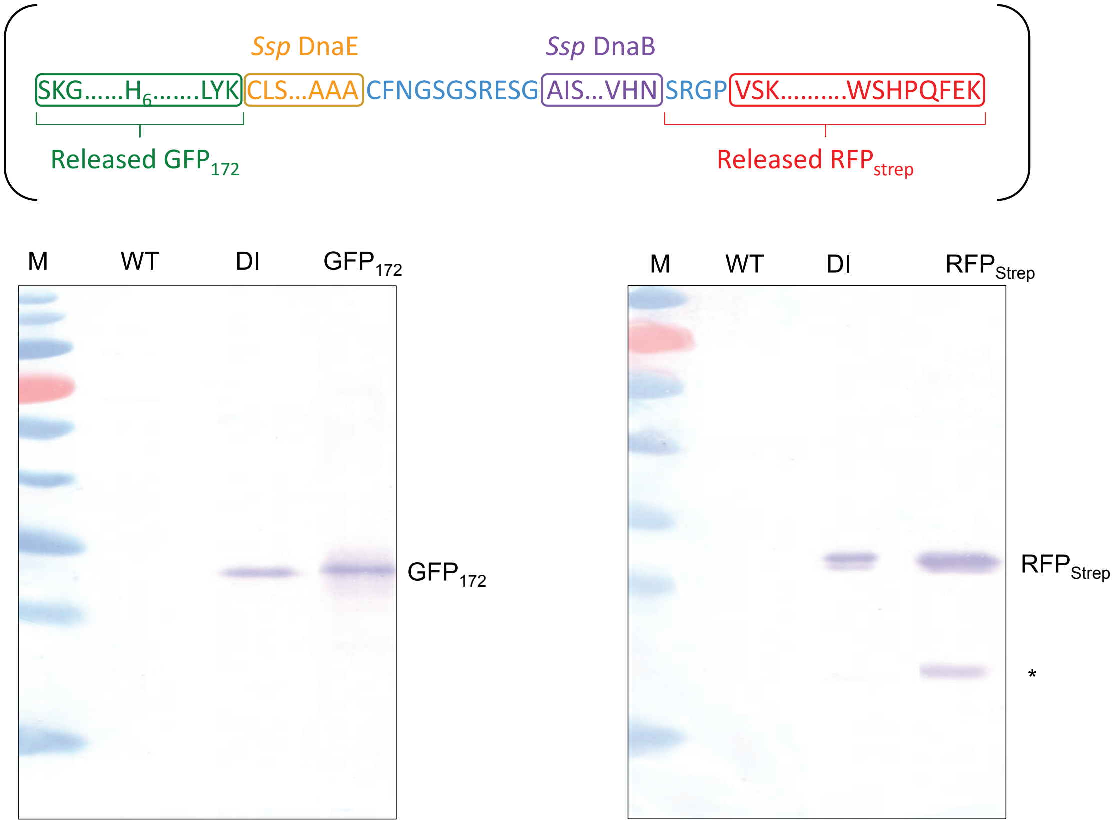

Supplementary Figure S3 | DI domain without the N-terminal linker extension preserves high autocleavage activity (cf. Figure 2 for DI containing the N-terminal linker extension sequence LEGGSKFAND). Cell extract of *E. coli* expressing the GFP_{172}-DI-RFP_{Strep} polyprotein containing a DI domain lacking the N-terminal linker extension between GFP_{172} and *Ssp* DnaE intein was analyzed using western blots probed with anti-GFP (left) and anti-Strep tag (right) antibodies, respectively.

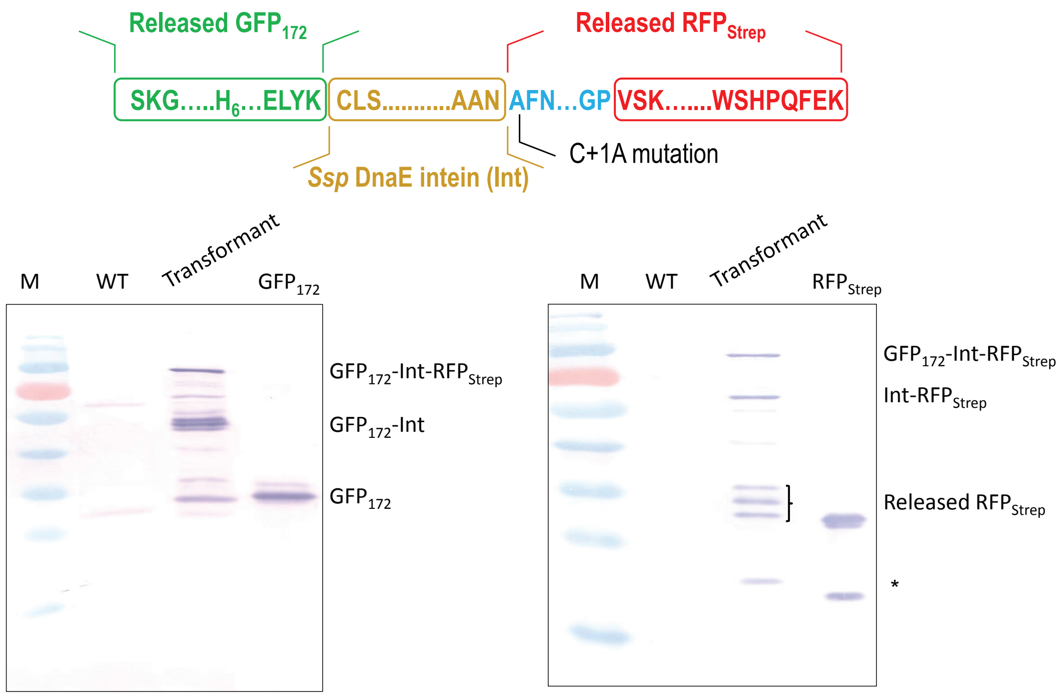

Supplementary Figure S4 | Inefficiency of single intein mediated polyprotein processing in *E. coli* (cf. Figure S3). A polyprotein vector containing a single *Ssp* DnaE intein domain was created and transformed into *E. coli* BL21(DE3). The *Ssp* DnaE intein domain has Asn at its C-terminus (as opposed to Ala in the DI domain), and the C+1 Cys is mutated to Ala. Protein extract of *E. coli* expressing the polyprotein coding sequence was analyzed using anti-GFP and anti-Strep tag western blots to detect release of the upstream and downstream proteins, respectively.

Supplementary Table S1 | Primers used for construct cloning in this study.

Name	Sequence
SalI-mGFP5	5'-ACGCGTCGACCAAGGAGATATAACAATGAGTAAAGGAG AAGAACT-3'
GFP-*XhoI*-R	5'-TGCCTCGAGGTGATGGTGATGGTGATGC-3'
mCherry-2A-F1	5'-GAGACGTCGAGTCCAACCCTGGGCCCGTGAGCAAGGGC GAGGAGGATAACATG-3'
mCherry-Strep	5'-CAAACTGAGGATGTGACCATCCAGAACCCTTGTACAGCT CGTCCATGCCGCCG-3'
GKZ-R	5'-CTGGAGCTCTCATTTTTCAAACTGAGGATGTGAC-3'
DnaB-*xbaI*-F	5'-TCTGGTTCTAGAGAGTCTGGAGCTATCTCTGGCGATAGT CTGATC-3'
DnaB-SRGP-R	5'-GCTCACGGGCCCGCGTGAGTTGTGTACAATGATGTCATT CGCGAC-3'
GFP-*KpnI*-F	5'-GACGGTACCCAAGGAGATATAACAATG-3'
mGFP5-His-*XhoI*-R	5'-AGTACTCGAGGTGATGGTGATGGTGATGACTTCCAATCC CAGCAGCTGTTACAAACTC-3'
KpnI-DnaE-F	5'-CGGGGTACCAAGTTTGCAAATGATTGTTTGTCC-3'
NdeI-G172-F	5'-GGAATTCCATATGAGTAAAGGAGAAGAACTTTT-3'
Nd172R	5'-GTGCCGCTTCATATGATCTGGGT-3'
Nd172F	5'-ACCCAGATCATATGAAGCGGCAC-3'
G172-*KpnI*-R	5'-GTGGTACCTTTGTATAGTTCATCCATG-3'
CAT-2A-F1	5'-GAGACGTCGAGTCCAACCCTGGGCCCGAGAAAAAAATC ACTGGATATACCAC-3'
CAT-STREP-R	5'-CAAACTGAGGATGTGACCATCCAGAACCCGCCCCGCCC TGCCACTCATCGCAG-3'
G172-DnaE-R	5'-AGGACAAACATTTGTATAGTTCATCCATGCC-3'
G172-DnaE-F	5'-ACTATACAAATGTTTGTCCTTCGGAACTGAG-3'
XBF	5'-CCCTCTAGAAATAATTTTGTTTAAC-3'
MBR	5'-CGCGGATCCCTTGTACAGCTCGTCCATGC-3'
GFS2	5'-GCACCGCGGGAAGTAAAGGAGAAGAACTTTTC-3'
GRS	5'-CGCGAGCTCTTATTTGTATAGTTCATCCATGC-3'

XhoI-Int-F	5'-CATCACCTCGAGGGAGGATCTAAGTTTGC-3'
DnaENAFN-*XbaI*-R	5'-AACTCTAGAACCAGAACCATTGAAAGCGTTTGCAGCAATAGCACCGTTTGC-3'
IDnaE-*KpnI*-F	5'-ACTCCTCGAGTATGCATTGTCCTTCGGAACTGAGATACTTAC-3'
DnaE-*XbaI*-R-2	5'-GTATTCTAGAACCACCAGCTGCAGCAATAGCACCGTTTGCGAG-3'
IDnaB-*ApaI*-R	5'-ATGGGCCCACTTCCCGCGTGTACAATGATGTCATTCG-3'
SacI-*Nhe*-DI-F3	5'-TATATGAGCTCTCTGGCTAACTAGAGAACCCACTGCTTACTGGCTTATCGAAA-3'
Strep-*NotI*-R	5'-TTTTCTTTTGCGGCCGCTCATTTTTCAAACTGAGGATG-3'

Supplementary Methods

Gel electrophoresis and western blot analysis. Protein extracts were denatured in SDS-PAGE sample buffer (50 mM Tris-HCl pH 6.8, 2% sodium dodecyl sulfate, 10% glycerol, 0.01% bromophenol blue and 25 mM 2-mercaptoethanol) by heating at 95°C for 5 min. SDS-PAGE and western blot of the denatured samples were carried out as described previously.[1] Anti-GFP rabbit serum (Invitrogen, Grand Island, NY), mouse anti-His tag (Lamda Biotech, St. Louis, MO) and rabbit anti-Strep tag II (Genscript, Piscataway, NJ) were used to detect processed proteins. Goat anti-rabbit or goat anti-mouse conjugated with alkaline phosphatase was used as secondary antibody (Southern Biotech, Birmingham, AL).

CAT assay. Extracts of *E. coli* expressing GFP_{172}-DI-CAT_{Strep} were diluted in a reaction mixture containing 100 mM Tris buffer (pH 7.8), 8 mM of 5,5'-Dithio-bis (2-Nitrobenzoic Acid) (DTNB), and 16 mM of Acetyl Coenzyme A. After equilibration, the reaction was initiated by adding 0.005% of chloramphenicol. The enzyme activity was calculated based on the increase of A_{412} over 5 min at room temperature.

References

1. Zhang, B., Rapolu, M., Huang, L. & Su, W. W. Coordinate expression of multiple proteins in plant cells by exploiting endogenous kex2p-like protease activity. *Plant Biotechnol. J.* **9**, 970-981 (2011).
